# 

**Published:** 1856-04

**Authors:** 


### CONTENTS OF NO. II, VOL. III.

ORIGINAL COMMUNICATIONS.

Page.

Practical Gleanings—By Prof. Allen 81

Extracts from the Valedictory Address, delivered at the commencemence of session of 1855–6, of Medical Department, Iowa University—By Prof. M'Gugin...... 89

Case of Labor, with spontaneous Evolutions, &c—By A. M. Carpenter, M. D.................. 99

Use of Iodide of Zinc—By Prof. M'Gugin..................... 103

DEPARTMENT OF SELECTIONS.

Progress of Physiology. From the German—By W. Krause, M. D., Cincinnati.................. 104

On the faculties possessed by certain elements of the blood, of regenerating the vital properties....... 105

On the Secretion of Sugar in the Animal Economy—By Bernard Henry, M. D.................. 107

Severe Inflammatory Croup....... 109

Lecture on Fever—By Wm Stokes. 110

Epidemics—By S. H. Dickson, M D 115

Socialistic Theories in their Medical Aspect .................. 126

Page.

EDITORIAL DEPARTMENT.

Medical Progress. New Era...... 133

Legislative Interference in Medical Institutions.................. 140

Public Charities................. 146

Toadyism.............. ....... 148

A great benevolent enterprise..... 148

Exchanges..................... 149

Dr. Brainard's Introductory, again. 149

American Medical Association.... 150

Iowa State Medical Society....... 150

That Likeness.................. 150

Our Journal...... ............. 151

Organization of a Medical Society, Keokuk ........... .......... 153

College of Physicians and Surgeons of the Iowa University, Session of 1855–6..................... 153

BIBLIOGRAPHICAL NOTICES.

Columbus City Courier........... 156

Headland, on the Action of Medicines......................... 157

Outlines of Chemistry—By William Gregory, M. D................ 158

Sacramento Medical Society...... 158

Poetry from Pindar.............. 159

April Fooled... ................. 160

